# From perfusion to pathology: using echocardiography to characterize a vascular, cystic atrial myxoma

**DOI:** 10.1093/ehjcr/ytag254

**Published:** 2026-04-09

**Authors:** Tevin Browne, Theodore Velissaris, Eunice Onwordi

**Affiliations:** University of Southampton, School of Medicine, University Road, Southampton SO17 1BJ, UK; Cardiothoracic Unit, University Hospital Southampton NHS Foundation Trust, Tremona Road, Southampton SO16 6YD, UK; Cardiothoracic Unit, University Hospital Southampton NHS Foundation Trust, Tremona Road, Southampton SO16 6YD, UK; Cardiothoracic Unit, University Hospital Southampton NHS Foundation Trust, Tremona Road, Southampton SO16 6YD, UK

**Keywords:** Advanced echocardiography, Cardiac masses, Multiplanar reconstruction

**Figure:**
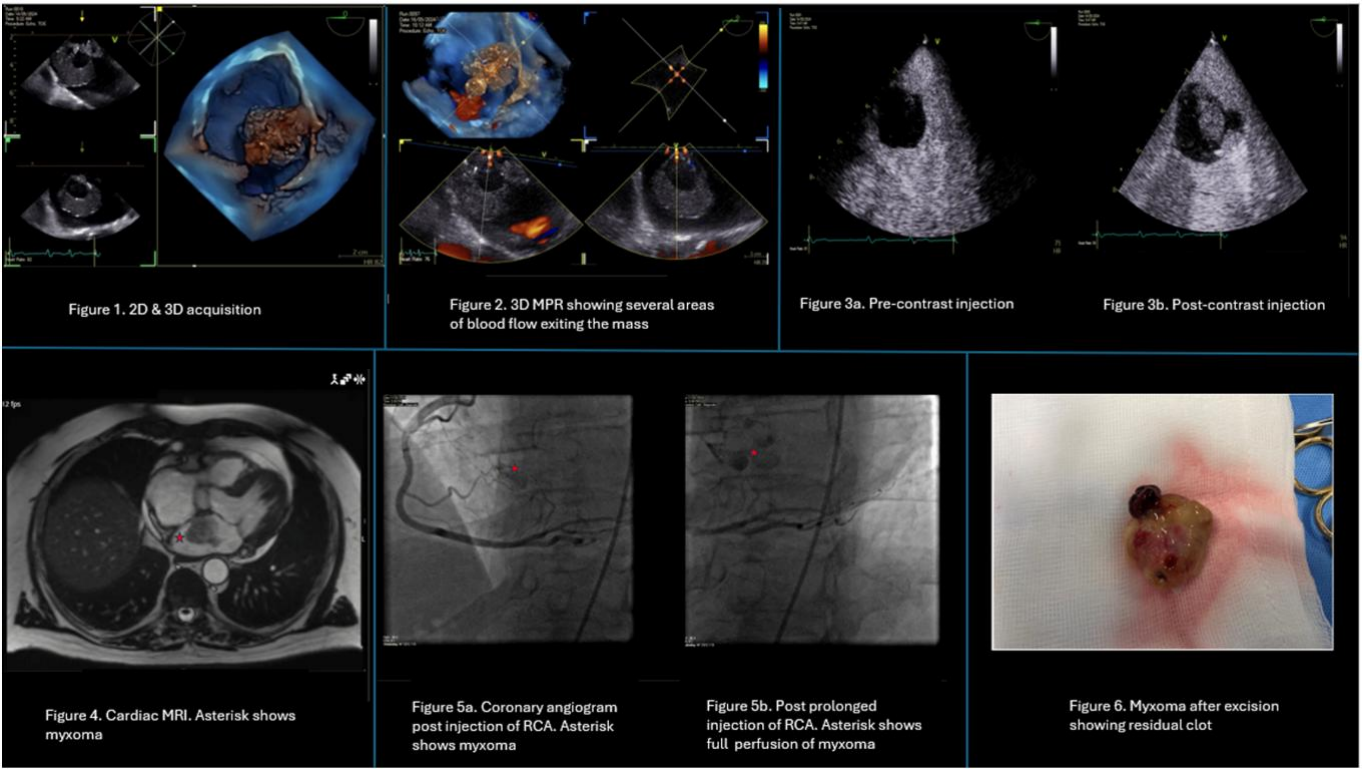


## Summary

Primary cardiac tumours are rare, and although atrial myxomas represent the most common primary cardiac tumour in adults, cystic or haemorrhagic variants are exceptionally uncommon.^1,2^ Current ESC guidelines endorse echocardiography as the first-line investigation for cardiac masses; this case illustrates the additional diagnostic value of advanced echocardiography in pre-operative characterization.^3^

## Case description

An 80-year-old man was referred with a 5-month history of exertional dyspnoea and presyncope. His medical history included rheumatic fever, atrial fibrillation, and type 2 diabetes. Transthoracic echocardiography demonstrated a mobile, irregular left atrial mass with a central echolucent component attached to the interatrial septum. In line with guideline-recommended multimodality assessment, transoesophageal echocardiography (TOE) was performed using a GE Vivid platform.

Two-dimensional TOE confirmed a 3.69 × 3.72 cm left atrial mass. Three-dimensional datasets were acquired over four cardiac cycles using focused 4D zoom acquisition from the mid-oesophageal four-chamber view, with the region of interest centred on the atrial mass and mitral valve. Orthogonal imaging planes were optimized to define tumour margins and septal attachment. The reconstructed volume was rotated to obtain a left atrial ‘surgeon’s view’. Multiplanar reconstruction demonstrated intra-tumoural vascular flow exiting the lesion on colour Doppler and delineated a pedunculated attachment to the fossa ovalis without mitral valve involvement, supporting the feasibility of complete *en bloc* excision. Contrast-enhanced TOE using SonoVue demonstrated intense contrast uptake, indicating marked vascularity.

Cardiac magnetic resonance imaging confirmed a bilobed left atrial mass. Tissue characterization demonstrated intermediate T1 and high T2 signal intensity with avid central enhancement and persistent peripheral hypoperfusion on first-pass and late gadolinium imaging, consistent with a vascularized atrial myxoma. Cardiovascular magnetic resonance (CMR) excluded extracardiac extension and confirmed preserved ventricular function without myocardial scarring. Coronary angiography demonstrated arterial supply from the right coronary artery with contrast opacification of the tumour during prolonged injection. Significant distal left main coronary artery disease with moderate proximal left anterior descending involvement was also identified.

The patient underwent surgical excision of the mass with concomitant coronary artery bypass grafting for significant left main coronary artery disease and left atrial appendage occlusion for atrial fibrillation. Histopathology confirmed a myxoma with extensive haemorrhage, haemosiderin deposition, and prominent vascularity.

This case demonstrates that advanced echocardiographic techniques, including 3D multiplanar reconstruction and contrast imaging, provide real-time assessment of tumour morphology and vascularity, supporting timely surgical planning and complementing multimodality imaging.

## Supplementary Material

ytag254_Supplementary_Data

## Data Availability

Data sharing is not applicable to this article as no new datasets were generated or analysed.
